# Antiviral drug research for Japanese encephalitis: an updated review

**DOI:** 10.1007/s43440-022-00355-2

**Published:** 2022-02-19

**Authors:** Shaun Joe, Abdul Ajees Abdul Salam, Ujjwal Neogi, Naren Babu N, Piya Paul Mudgal

**Affiliations:** 1 Manipal Institute of Virology, Manipal Academy of Higher Education, Manipal, Karnataka 576104 India; 2 Department of Atomic and Molecular Physics, Centre for Applied Nano Sciences, Manipal Academy of Higher Education, Manipal, Karnataka 576104 India; 3 Division of Clinical Microbiology, Department of Laboratory Medicine, Karolinska Institute, ANA Futura, Stockholm, Sweden

**Keywords:** Antiviral, Drug targets, In-silico molecular modeling, Japanese encephalitis virus, Nucleic acid-based antiviral, Replication cycle-based antiviral Screening

## Abstract

Japanese encephalitis (JE) caused by the Japanese encephalitis virus (JEV) is one of Asia's most common viral encephalitis. JEV is a flavivirus, common in rural and sub-urban regions of Asian countries. Although only 1% of JEV-infected individuals develop JE, there is a 20–30% chance of death among these individuals and possible neurological sequelae post-infection. No licensed anti-JE drugs are currently available, despite extensive efforts to develop them. Literature search was performed using databases such as PubMed Central, Google Scholar, Wiley Online Library, etc. using keywords such as Japanese encephalitis virus, antiviral drugs, antiviral drug screening, antiviral drug targets, etc. From around 230 papers/abstracts and research reviews retrieved and reviewed for this study, approximately 180 most relevant and important ones have been cited. Different approaches in drug testing and various antiviral drug targets explored so far have been thoroughly searched from the literature and compiled, besides addressing the future perspectives of the antiviral drug development strategies. Although the development of effective anti-JE drugs is an urgent issue, only supportive care is currently available. Recent advancements in understanding the biology of infection and new drug targets have been promising improvements. Despite hindrances such as the unavailability of a proper drug delivery system or a treatment regimen irrespective of the stage of infection, several promising anti-JE candidate molecules are in different phases of clinical trials. Nonetheless, efficient therapy against JEV is expected to be achieved with drug combinations and a highly targeted drug delivery system soon.

**Graphical abstract:**

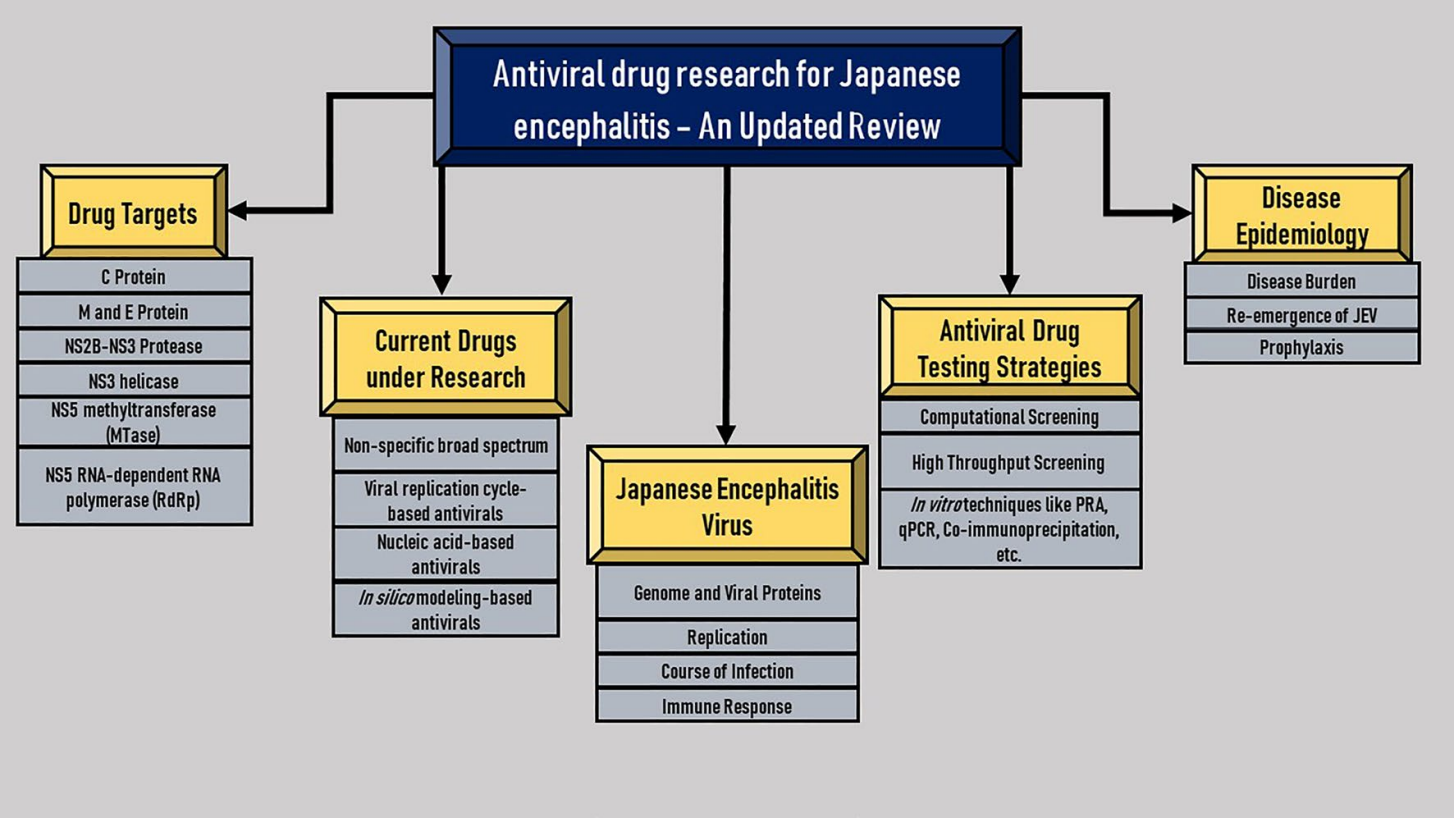

## Introduction

Japanese encephalitis (JE) caused by the *Flavivirus,* Japanese encephalitis virus (JEV), is the most common viral encephalitis in Asia. Although rare, it has also been reported from northern Australia and western pacific regions [1]. JEV has a single-stranded RNA genome and is primarily transmitted by mosquitoes, *Culex vishnuii*, and *Culex tritaeniorhynchus* [2]. Ardeid birds (herons, egrets), along with bats, serve as the primary virus reservoirs. Pigs are the most common amplification hosts for JEV, wherein the virus amplifies, resulting in a very high viral titer. A significant risk factor that facilitates the transmission of JEV to humans is pig rearing. Vector-borne transmission of the virus to humans and domestic animals results in the further spread of JEV. JEV is not transmitted from person to person or from domestic animals such as horses, making humans and horses dead-end hosts [3]. Humans and horses can develop severe symptoms, commonly encephalitis, whereas pigs rarely show any clinical manifestation of the infection. JEV-induced encephalitis has a mortality rate as high as 25–30%, and up to 50% of surviving patients suffer from neuropsychiatric sequelae [4].

Effective antiviral therapy for JEV is of great importance, owing to the enzootic nature of the virus. This characteristic of the virus enables it to persist in nature to the extent that it is never entirely eradicated from the environment [5]. With the advent of modern technologies, research in antiviral drug development for JE has seen a sturdy increase in the last few decades. Making a therapeutic drug readily available to the JEV risk groups at an affordable cost is the primary aim of the JEV antiviral research [5, 6].

This review discusses the current strategies and approaches in antiviral drug research against JEV and the recent trends in discovering virus-targeted compounds that can be potentially developed into therapeutic drugs.

### JEV genome and structure

**Fig. 1:**
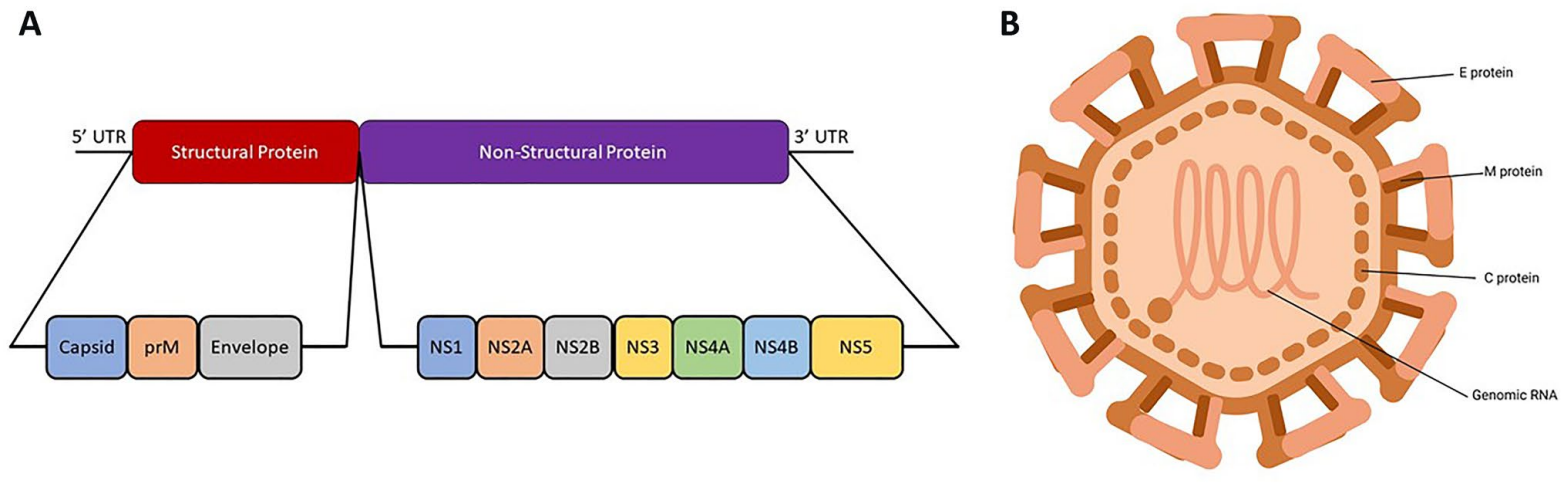
**A** Japanese encephalitis virus genome; **B** structure of Japanese encephalitis virus

JEV comprises a single-stranded, positive-sense RNA genome, ~ 11 kb in length. The genome has one open-reading frame (ORF) encoded between the 5′ and 3′ non-coding regions (NCRs) and lacks a poly-A tail at the 3′ end. The open-reading frame codes for a polyprotein precursor (~ 3432 amino acids), which gives rise to ten distinct proteins, comprising of the three structural (capsid, C; premembrane, M; and envelope, E) and the seven non-structural (NS1, NS2A, NS2B, NS3, NS4A, NS4B, and NS5) proteins [7, 8] (Fig. 1A). There are primarily four proteases involved in the site-specific cleavage of the polyprotein to yield the functional proteins (Table 1).

**Table 1:** Proteases involved in viral replication

Protease	Origin	Function
Signal peptidase	Host protease	Cleaves at the C-M, M-E, E-NS1, and NS4A-NS4B junctions [7–9]
Protease NS3-NS2B	Viral protease	Cleaves at the NS2A-NS2B, NS2B-NS3, NS3-NS4A, and NS4B-NS5 junctions Internal sites within the C and NS4A proteins [7–9]
Furin-like protease	Host protease	Cleaves precursor M (prM) to M [7–9]
Protease enzyme	Host protease	Cleaves at the NS1-NS2A junction [7–9]

*C* capsid, *M* membrane, *E* envelope, *NS* non-structural, *prM* premembrane

#### Structural proteins

JEV is roughly spherical with a diameter of 510 Å. It is composed of three structural proteins and an outer lipid bilayer membrane. The E and M proteins protrude from the lipid membrane and are anchored through transmembrane helices. The viral capsid is disordered and encloses the viral genome (Fig. 1B). It plays a pivotal role in viral replication and nucleocapsid formation. The precursor M protein (prM) is involved in folding the E protein. It acts as a chaperon and prevents the oligomeric rearrangement of E proteins triggered by the low pH of the endosome. The E protein activates membrane fusion by binding to the virus-specific cellular receptor and highly immunogenic protein. It is also essential for the entry of the virion into the cells, protein assembly, and budding [8–11].

#### Non-structural proteins

The exact function of NS1 protein remains unclear, although it is understood to be involved in the replication step as it localizes with the double-stranded RNA (dsRNA) [12]. The flavivirus NS2A protein is essential for genome synthesis and assembly. The NS2B protein complex with the NS3 protein exhibits serine protease activity at the N-terminal [10, 13]. The C-terminal region of NS3 protein has helicase activity [8, 10].

NS5, just like NS3, is a multi-enzymatic protein with a guanylyltransferase/methyltransferase domain in the N-terminal region and an RNA-dependent RNA polymerase domain in the C-terminal. The interaction of NS3 and NS5 is of utmost importance during the replication cycle. The hydrophobic NS4A coordinates with NS1 during replication; however, the function served by this alignment is unclear. Similarly, the role of flavivirus NS4B protein is hardly known, although NS4B has been also found to co-localize with dsRNA. Guanylyltransferase/methyltransferase domain of the NS5 protein is responsible for the 5′ capping of the genomic RNA, and the RNA-dependent RNA polymerase domain mediates RNA replication [8, 10, 13–15].

##### Replication

**Fig. 2:**
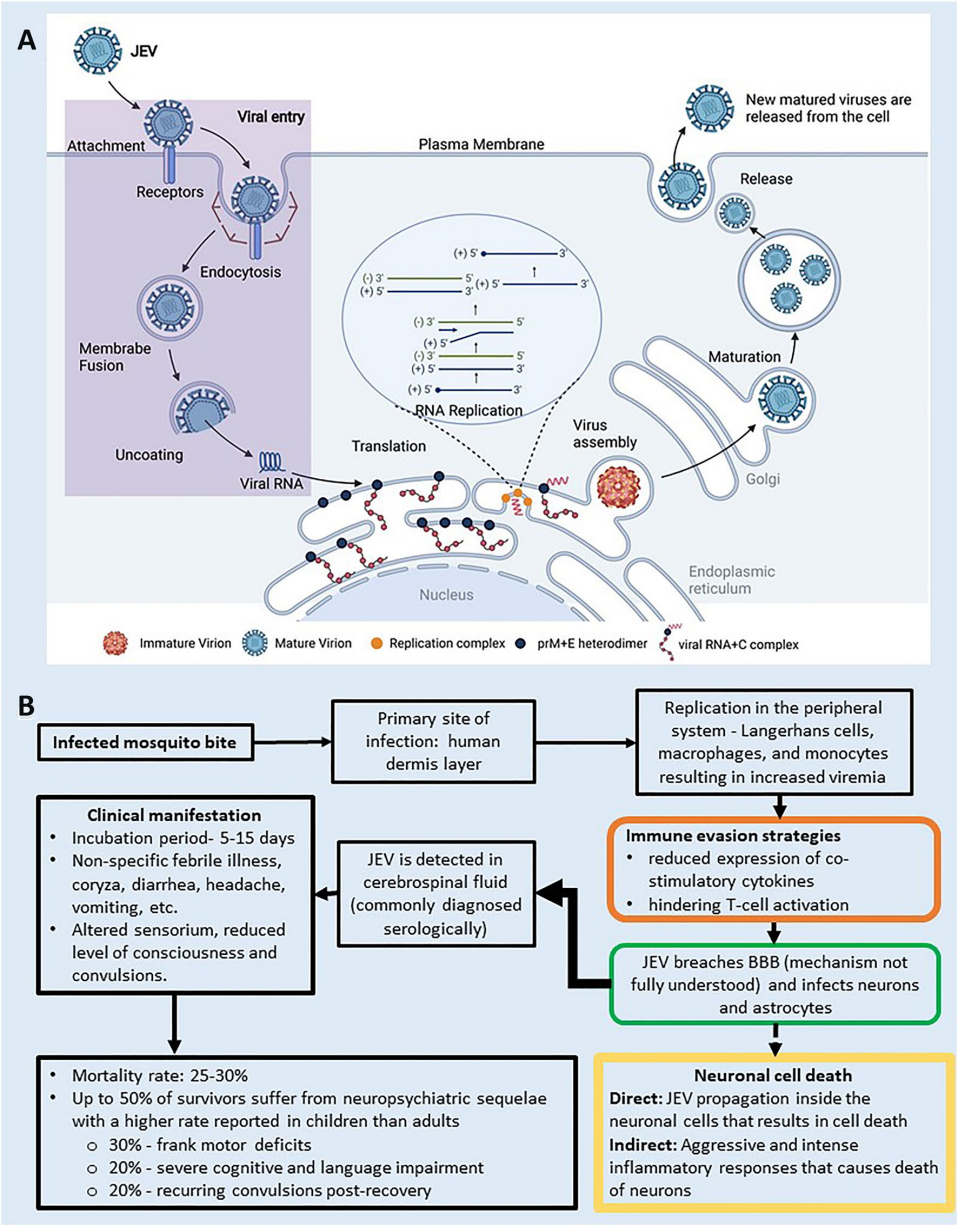
**A** Japanese encephalitis virus replication [16, 17]; **B** the progress of Japanese encephalitis virus infection

JEV is a *flavivirus* in which the nucleocapsid is enclosed by a lipid bilayer composed of the membrane-anchored M and E proteins. JEV, along with the other *flaviviruses*, shares a similar replication mechanism. Viral entry is an active process involving several complex interactions between the virus and the host cell. These non-specific binding of the E protein to cellular receptors like heparin sulfate, on the cell surface, further facilitates more specific interactions [13] (Fig. 2A). Cellular receptors like PLVAP (plasmalemma vesicle-associated protein) and GKN3 (gastrokine3) promote clathrin-dependent or clathrin-independent endosome formation [8, 14]. The low pH inside the endosome induces conformational changes in the E protein, triggering the fusion of the viral membrane with the inner wall of the endosomal membrane. The genome is released into the cytoplasm immediately after the fusion of the membranes and is translated into the precursor polyproteins [8, 13, 14].

In the cytoplasm, the precursor polyproteins are translated from the genome and are cleaved into two precursor polyproteins that are further processed to generate the three structural and other non-structural proteins [7, 8, 11]. The non-structural proteins, along with host factors, are involved in the viral replication that occurs in the viroplasm. Viroplasm is an ER-derived membranous organelle housing the replication complexes and is the site of viral RNA synthesis. NS3 and NS5 catalyze the replication step and coordinate their multiple enzymatic activities to facilitate RNA synthesis, 5′ capping, and methylation [8, 11, 18]. The C protein complexed with the newly synthesized genomic RNA is enveloped on the ER membrane with two viral glycoproteins (prM and E). This step results in the formation of the immature virion composed of 60 protruding spikes made of prM and E heterodimers. The constitutive secretory pathway followed by furin-mediated cleavage of the prM protein to M in the trans-Golgi network results in viral maturation. The mature virions (~ 50 nm diameter), composed of 30 densely packed rafts of hetero-tetramers made of E and M proteins, are released by budding [13]. The progress of infection and its development to Japanese encephalitis is illustrated in Fig. 2B [19–22].

### Disease burden

#### Global outlook

From as early as the 1870s, frequent outbreaks of encephalitis have been reported from Japan. Significant epidemics were recorded every 10 years, with occasional peaks of encephalitis cases occurring during the summer season. These cases of encephalitis were then called ‘type B encephalitis’ to distinguish it from von Economo’s encephalitis clearly. In 1935, the Nakayama strain of JEV was first isolated from an infected patient, and the virus was named ‘Japanese encephalitis virus’. The virus was then classified into the genus *Flavivirus* (family—*Flaviviridae*) [6]. Flaviviruses comprise over 70 different virus species, and phylogenetic analysis suggests that JEV evolved from an African ancestral virus. The present circulating strain of JEV was traced to have evolved from the ancestral strain in the Indonesian-Malaysian region. The virus was then distributed across Southern and eastern parts of Asia and Pacific regions over time [23]. Rapid urbanization, population explosion, climate change, globalization, and deforestation have resulted in the spread of the virus from one area to the other [6]. Today, up to 60% of the world’s population is inhabiting JE-endemic region. Approximately 67,900 cases of JE occur annually, of which only 10% of cases are reported to the World Health Organization [1]. Around 50% of these cases occur in China, and approximately 75% affected are children aged 0–14. These are some of the aspects, which make JE one of the most significant viral encephalitis globally [1]. The WHO published the number of JEV cases reported from different countries around the world. These data were published in 2020 and were focused on vaccine-preventable diseases and immunization monitoring [24]. The countries which recorded the maximum number of JE cases over the years are discussed below.

#### Distribution around the world

##### China

**Fig. 3:**
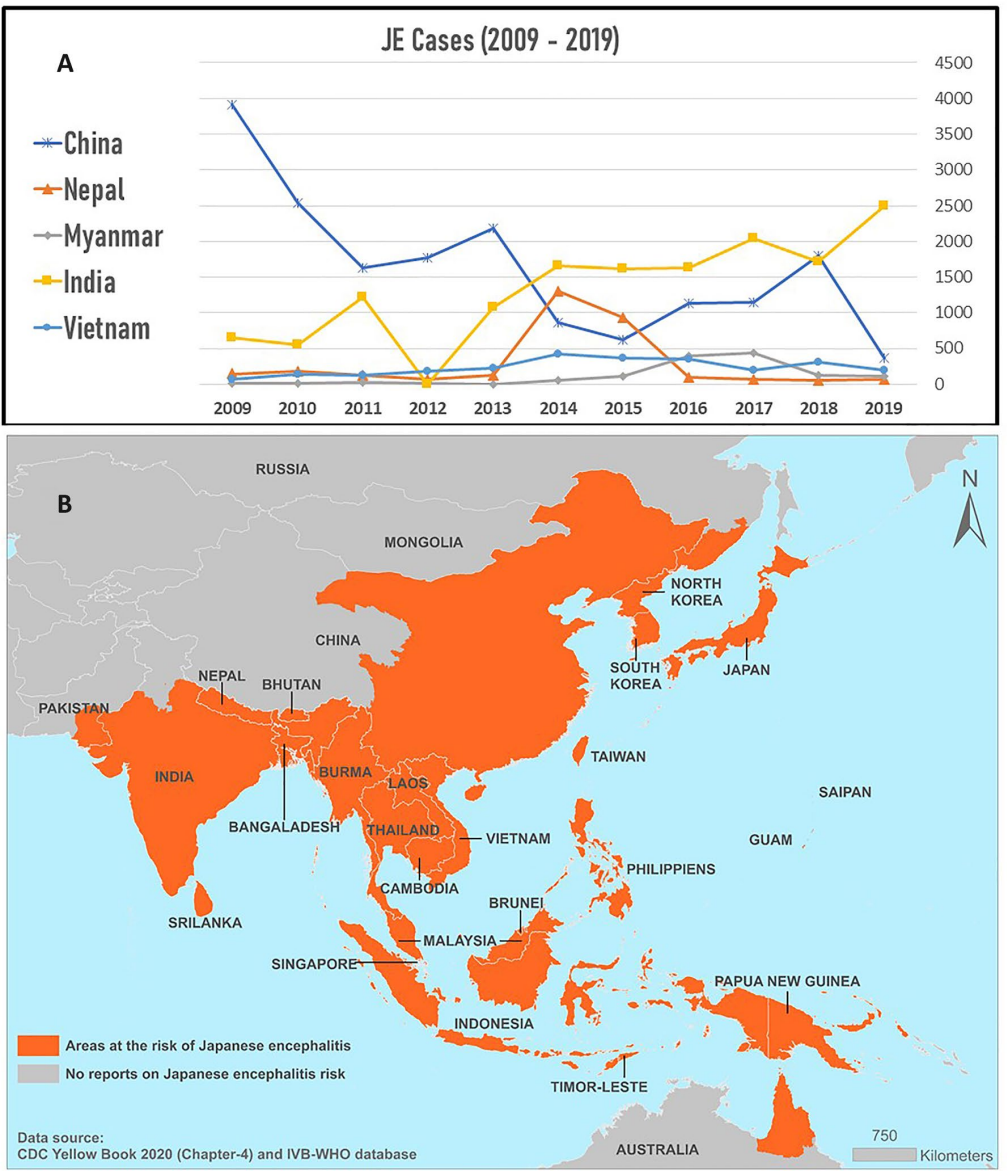
**A** JE cases from 2009 to 2019 in China, Nepal, Myanmar, India, and Vietnam. These countries are endemic to JEV and contribute towards a majority of the overall cases reported around the world; **B** geographic distribution of Japanese encephalitis virus (JEV). The JEV distribution map was created using ArcGIS v.10.4 (ESRI, Redlands, CA, USA)

Chinese mainland records a high prevalence of JEV and is the foremost region of JEV endemism. China has an efficient and sensitive system to trace each JE case annually. This case reporting system was established in 1951, and has been an integral part of managing and mitigating various spikes and outbreaks of JEV. Although cases are reported throughout the year, they significantly rise from June to October. The number of morbidities from these months’ accounts for 97% of the overall cases in mainland China. Two major JE epidemics have been reported in China—1966 and 1971 [25–27]. The overall trend in the number of JEV cases in China from 2009 to 2019 is represented in Fig. 3A [24].

##### India

India’s first case of JEV was recorded in 1955 and was first isolated in 1958. Until the early 1970s, JE was reported only from southern India but received nationwide attention after the significant outbreak in 1973 from the Bankura district of West Bengal with a 42.6% case-fatality rate. Subsequently, several cases of encephalitis were reported from different parts of the country [28]. India’s biggest outbreak of Japanese encephalitis in recent history occurred in 2005 in northern India. The outbreak started with several cases reported from the state of Uttar Pradesh in July 2005, which escalated to nearly 5000 cases, mounting up to 1300 deaths by November 2005 [29]. Presently, there are 1000–2500 cases reported annually from India. These numbers are based on the total cases reported around the country, as collated in the National Vector Borne Disease Control and Prevention (NVBDCP) database. The actual disease burden of JE is expected to be higher in both the Indian as well as global context due to the several unreported cases [30]. India has seen a stable increase in JE cases over the years, as depicted in Fig. 3A [24].

##### Myanmar

Myanmar (formerly Burma) is a South Asian country endemic to JEV. A significant surge in the number of JE cases was first recorded in the Shan State, Myanmar, in 1974. The WHO has recorded an average of 118 cases per year. 2016 saw an increase in JE cases, which peaked by August 2017 in Myanmar. A steady decline was observed in the JE cases in Myanmar after 2017 due to several interventions such as control measures, vaccination programs, appropriate preparedness, and response (Fig. 3A) [24, 31].

##### Nepal

Nepal is a landlocked country sharing its borders with China and India. JE was first confirmed in the year 1978 and has been recorded steadily since. The surveillance of JE cases in Nepal is being monitored since 2004 by the World Health Organization (WHO) through a network of national vaccine-preventable diseases [32]. An average of 235 cases is recorded every year with a spike observed in 2014 (Fig. 3A) [24].

##### Vietnam

First isolated as early as 1951, JE is one of Vietnam's chief public health problems. Due to insufficient laboratory testing capabilities, acute encephalitis, an essential manifestation of JE, is considered a marker and reported for JE surveillance. Over recent years, an average of 236 cases per year has been recorded, with a significant increase in 2014–2016 (Fig. 3A). Substantial measures like nationwide vaccination programs, vector control, etc. have successfully brought down the number of cases [24, 33].

#### Epidemiology

The fatality of JE is as high as 20–30%, and a staggering 30–50% of survivors develop substantial neurologic sequelae. JE primarily affects children, but all age groups may be affected. Post-infection, most individuals develop natural immunity [1]. Two distinctive patterns of JEV cases have been observed from the tropical and temperate regions of JE-endemic countries. Significantly, large epidemics occur during the summer season in temperate areas like Nepal, China, Japan, the Korean peninsula, and northern India. On the contrary, although case distribution is sporadic during the rainy season, the cases peaks in the tropical regions, particularly the southern part of Vietnam, Thailand, Indonesia, and Sri Lanka [7, 34].

There are five different genotypes of JEV (namely I, II, III, IV, and V) based on the gene sequence of the E protein [35]. The difference in the virulence of each genotype in humans is debated. Genotype III has a more widespread geographical distribution than the other four genotypes. A motley collection of ecological, environmental, climatic, and human behavioral factors like irrigation schemes, development of the rice industry, etc., have resulted in the virus spread [29]. Figure 3B summarizes the geographic distribution of JEV.

#### Re-emergence of JEV

There are primarily two factors that account for the re-emergence of JEV. First, there has been an unprecedented and rapid surge in the population of the JE-endemic countries in the past decades. One such example of the population increase was observed in the endemic Asian regions. The population of around 1.7 billion in the mid-twentieth century almost doubled to around 3.5 billion by the early 2000s. Second, the escalation of pig rearing for food, coupled with the development of rice-production systems, which fueled rice farming and cropping intensity, also contributed to the emergence of JEV. An example to quote in this context would be an increase in pork production in China, which doubled from 1990 to 2005 [29, 34].

#### Prophylaxis

**Fig. 4:**
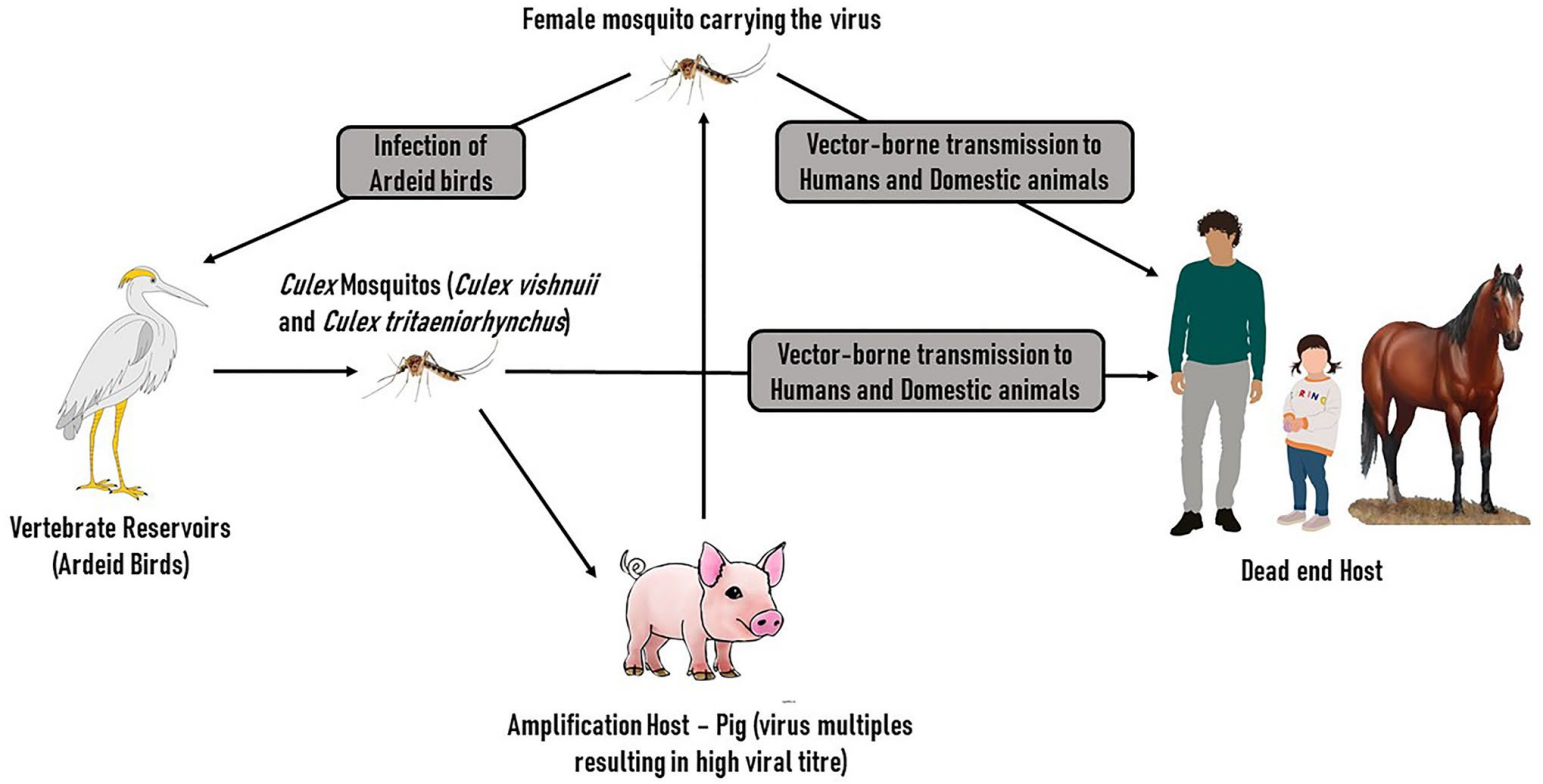
The life cycle of Japanese Encephalitis Virus. Ardeid birds (pond herons, egrets) serve as virus reservoirs causing the enzootic nature of the virus. Culex mosquitoes that feed on a viraemic host get infected, and actively transmit the virus to other hosts after 9–12 days of incubation. Pigs often act as amplification hosts and do not manifest any significant symptoms of the infection. They are the primary host of the virus. The vector-borne transmission of the virus to man and domestic animals leads to a severe disease condition. The absence of person-to-person transmission makes man a “dead-end” host

Prophylaxis is the best way to prevent JEV infection. The control of *Culex* mosquitoes, the primary vectors for JEV, is crucial for prevention. Figure 4 shows the life cycle of JEV. Ideal breeding grounds for mosquitoes, such as paddy fields, help spread the virus by attracting migratory birds. Control measures in paddy fields may include the use of larvicides and larvivores such as guppy fishes (*Poecilia reticulata*) [6]. Close monitoring of animals is vital in pig rearing to prevent pigs' infection. Vaccination of these animals is also a logical way to break the transmission of JEV to pigs [36, 37].

#### Limitations of prophylaxis

The most effective method to prevent JE would be the selective vaccination of risk groups. The discovery of the live attenuated vaccine for JEV has proved efficient in preventing JE [38]. Although the vaccine requires multiple doses, it is relatively safe and effective [6]. There has been no other variable that significantly reduced the JE disease burden, and therefore, prompt immunization is prioritized over mosquito control and life stock management. Vaccination is highly recommended in places suitable for JEV transmission even if the reported JE cases are fewer. WHO recommends that all travelers to JEV endemic regions take precautions and get vaccinated to reduce the risk for JE [10].

Currently, various inactivated vaccines (mouse brain-derived, Vero cell-derived) and live vaccines (attenuated and recombinant (chimeric)) are in use. Among vaccine candidates in the late clinical trial phases, a live attenuated, yellow fever virus-based chimeric vaccine developed in Vero cells is the most promising candidate. A single dose has shown to be highly productive. Live attenuated vaccine made from the SA14-14–2 virus strain is the most widely used vaccine in JE-endemic areas and has 99.3% efficacy. The single dose of the vaccine was most efficacious when administered days/weeks before possible exposure to infection [7, 8, 10].

The most challenging task is rendering the vaccine available to the poor and rural communities, considering the compliance and delivery costs. Imprecise estimation of JEV disease burden in certain areas due to the absence of efficient surveillance infrastructure reduces the vaccine's reach. Public health interventions often focus on control measures over JEV immunization due to the vaccine's enormous cost and multiple-dose regimen [8, 10]. All these evidence points towards the significance of developing an efficient antiviral drug for JE treatment.

### Current therapeutic strategies

Current treatment strategies for JEV infection mainly involve supportive care and are not targeted at attenuating the virus [6]. A study investigating the use of ribavirin (a broad-spectrum antiviral) against JEV by controlled clinical trials showed that ribavirin has a more negligible effect on treating JEV [39]. A naturally occurring compound called ‘rosmarinic acid’, found in various *Labiatae* herbs, reported to have antiviral activity against *Flaviviruses,* was used for preclinical studies. This compound reduced viral replication of JEV (GP78 strain) in mice brains [40]. Curcumin is another compound shown to have antiviral activity against JEV in an in-vitro study. This compound reduced cellular reactive oxygen species level and maintained cell membrane integrity, thus preventing cell death. It was also observed that curcumin reduced apoptotic signaling molecules and stress-related proteins [41]. Minocycline, a derivative of the tetracycline group of antibiotics, showed remarkable results as an antiviral drug for JEV. An in-vivo study exploring its potential as a possible anti-JEV drug also showed promising results. Minocycline reduced viral titer significantly and prevented neuronal apoptosis and microglial activation [42]. The blood–brain barrier, which becomes impaired during the infection, was protected due to minocycline effects [43].

The budding of dengue type II (DEN-2) and JEV through the endoplasmic reticulum can be hindered by glucosidase inhibitors like N-nonyl-deoxynojirimycin (*N*N-DNJ). *N*N-DNJ is an imino sugar derivative that interrupts virus development by blocking the trimming step of N-linked glycosylation, thus causing misfolding of viral protein [44]. Another novel approach targeted the 3′ non-coding region of JEV using a synthetic oligonucleotide-based DNAzyme, which successfully inhibited virus replication in-vitro as well as in a mouse model [45].

Several approved antivirals have been repurposed to check for activity against JEV. These studies were focused on specific drug targets and were studied by high-throughput screening, computational methods, etc. Calcium inhibitors such as manidipine, cilnidipine, and benidipine hydrochloride inhibited virus infection at either entry or replication and even during budding [46]. Although these compounds were promising and had high efficacy in in-vitro or in-vivo systems, they failed to reproduce similar effectiveness in human trials or were found unsuitable for use in a clinical study. A placebo-controlled clinical trial with interferon-alpha-2a, in confirmed JE cases in children, remains the only study to date effective against JEV. Unfortunately, after 3 months of their discharge, a survey proved the inefficiency of interferon-alpha-2a in treating JE [47].

## Methods

Research papers, review articles, and books related to JEV and JE, including its transmission and replication cycle, global distribution, disease dynamics, and immune responses, were searched in PubMed Central, Google Scholar, Wiley Online Library, etc. Surveillance data on the recent number of JEV cases in various countries worldwide were retrieved from the WHO and government websites like NVBDCP. Keywords, such as Japanese encephalitis virus, antiviral drugs, antiviral drug screening, etc., were primarily used for the search. Studies published until September 2021 on antiviral drug research against JEV, screening strategies, clinical and laboratory trials, and viral targets were collected for this review. The review focuses on the current disease burden of JEV despite the availability of efficient vaccines, the current research trends, the available viral targets, and the widely studied potential antiviral drugs.

Approximately 230 papers/abstracts and review articles were retrieved and reviewed for this work. Various drugs studied for their potential anti-JEV activity were screened and collected to form a database. The drugs were sorted into three categories within the database after considering their targets, specificity, mode of action, etc. The various strategies employed to examine the antiviral activity of these drugs were also compiled. The different viral proteins and the potential therapeutic targets were also explored. The developments in recent years in understanding the biology of infection and the molecular mechanisms of viral replication were also discussed. This article is based on previously conducted studies and does not contain any new studies with human participants or animals performed by any authors.

## Viral targets and the course of infection

### Pathogenesis

The incubation period of JEV ranges between 5 and 15 days. The number of factors, such as route of entry, the virulence of the virus, genetic make-up, age of the host, etc., determine whether the infection progresses to JE [29]. The first step after the bite of an infected mosquito is the local replication of the virus in the skin, followed by transportation of the virus to the regional lymph nodes. The virus amplifies in the peripheral system (Langerhans cells, macrophages, and monocytes), resulting in high viremia, followed by the central nervous system (CNS). The virus is embedded in the connective tissue and other tissue during this amplification period. The clinical symptoms are entirely dependent on the invasion of the CNS from the blood [20, 21, 28].

If the virus can access susceptible neural cells of the CNS, it can lead to encephalitis. However, the infection of the non-neural tissues results in asymptomatic cases. Therefore, it is crucial to understand the mechanism of virus penetration to the CNS in comprehending the pathogenesis of viral diseases [48]. Although the process by which JEV crosses the blood–brain barrier is unknown, human post-mortem studies suggest a hematogenous route of entry [49]. Other risk factors, such as dementia, stroke, and sepsis, have been implicated in increasing the chance of neuro-invasion [28].

### Immune response

#### Innate immune response

Immediately after the entry of the virus, the host cells interacting with the virus start the production of many cytokines like type-1 interferons (TNF-α and interferon-γ). These cytokines induce an inflammatory response that inhibits virus replication. In addition to this, IFN-α and -β trigger lytic activity in NK cells and kill virally infected cells. IL-2 produced early during the viral infection enhances the lytic activity of NK cells. The activation of macrophages follows this initial activity of NK cells by IFN-γ, which expresses MHC class II molecules facilitating microbicidal action [20, 21, 43].

Granular lymphocytes in the bloodstream further attack the virus-infected cells and destroy them by phagocytosis. These cells are drawn to the entry site by chemoattractant molecules released by the complement system through a multicomponent enzyme cascade [20, 49]. Corticosteroids and anti-inflammatory drugs have been investigated in the treatment of JE [5]. Arctigenin (AR), a naturally occurring plant compound, strongly inhibited TNF-alpha production and reduced the inflammatory responses in an in-vivo study, which established its implications in being used to treat inflammation-related complications [50–52].

#### Adaptive immune response

The adaptive immune response is highly specific and can identify diverse pathogens. It displays immunologic memory and recognizes the self from foreign particles. The antigens are recognized by antibodies, after which they are cross-checked with surface receptors of immune cells, and signal molecules are secreted [20, 51, 52]. Lymphocytes and APCs are involved in adaptive immunity. Antigen sensitized B-lymphocytes are converted to effector plasma cells that produce antigen-specific antibodies. They clonally expand and secrete hundreds of antibody molecules that help virus neutralization. They also function as an essential effector molecule of humoral immunity [49, 51, 52].

JEV has several immune evasion strategies, among which the most important is by continuously altering their antigenic epitopes to transform themselves into new locally adapted quasispecies [53]. These new strains are often more virulent and are capable of causing severe infection. Thus, through significant mutations, the virus modifies itself and proceeds to be neurovirulent as they migrate to the CNS. The absence of protective immunity against newly emerged strains can result in the development of viral encephalitis. Thus, the humoral immune response plays a crucial role in protecting against JEV[20, 51, 53, 54].

#### Cell-mediated immune response

Along with the humoral immune response, cell-mediated immunity plays a vital role in clearing up virus-infected cells. It is executed primarily by IFN-γ-mediated-T helper or cytotoxic T-cell activity [51, 54, 55].

### Antiviral drug targets

Standard therapeutic compounds target the enzymes or receptors involved in essential viral functions. However, an alternative, complementary strategy is to focus on host cell factors like proteases as targets to arrest the development of the virus. Such an alternative approach reduces the likelihood of developing antiviral drug resistance and can target multiple viruses at a time. Despite this advantage, cytotoxicity and cellular side effects remain the significant drawbacks of targeting host cells [56, 57].

#### Potential JEV drug targets

**Fig. 5:**
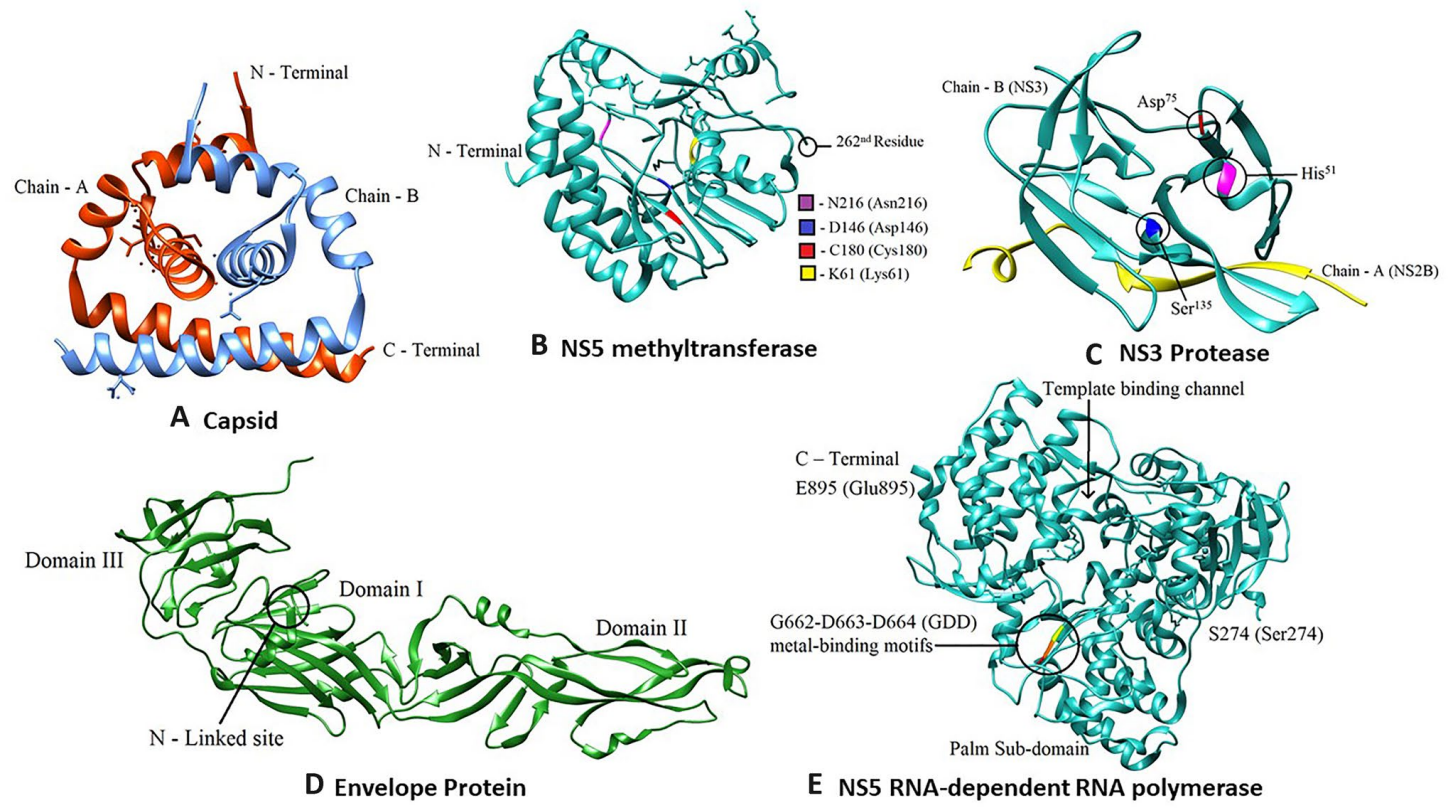
**A** JEV capsid dimer. Chain A is highlighted in orange whereas chain B is highlighted in blue, **B** NS5 methyltransferase (The N-terminal of the NS5 protein: residues 1–262 in cyan and active site, containing a catalytic K61-D146-C180-N216 motif in yellow, blue, red, and magenta, respectively, **C** Crystal structure of the JEV NS2B–NS3 protease (NS3 in cyan, NS2B in yellow, and the catalytic triad residues His51—Asp75—Ser135 in Magenta, Red, and Blue), **D** Crystal structure of JEV Envelope protein. JEV E protein possesses three domains characteristic of the flavivirus E protein, **E** JEV RdRp (G662-D663-D664 (GDD) metal-binding motifs are in red, orange, and yellow, respectively)

- **C protein** The basic 11 kDa C protein interacts with viral genomic RNA, forming the nucleocapsid (NC). The capsid folds into a dimer, in which each monomer contains four α-helices. The *N* and *C termini* contain charged residues, of which the *C-terminal* region may be involved with RNA association [58, 59]. The potential active site for binding the viral genome and nucleocapsid formation is the α4-4’ site on the dimeric interface due to its coiled-coil-like structure near the *C-terminal* (Fig. 5A) [58, 60]. The dimerization of C protein is a crucial step in its association with genomic RNA. Identifying compounds that block capsid dimerization or capsid–genome interaction can help develop effective anti-JEV drugs [61].
- **M and E proteins** The prM and E proteins are the main constituents of the immature virion, and this characteristic arrangement prevents their premature budding. With the help of cellular serine protease furin, the immature particles undergo conformational changes in the E protein, and this reaction facilitates maturation. Dimeric E protein is the major surface component of the immature virion whose conformational changes during maturation result in the mature virion's formation [59, 62]. The N-linked site in Domain 1 (D1) of the E protein has been associated with the infectivity of the virion and interaction with the cellular receptors (Fig. 5D). Therefore, the location and presentation of the glycan linked to N154 indicate that it is the binding site for the receptor (Fig. 5D) [59, 63, 64]. Studies involving the structural analysis of E protein revealed three potential drug targets: the β-OG ligand-binding pocket, E-protein rafts in the mature virus, and E homotrimers [59, 62, 65].
- **NS2B-NS3 protease** The serine protease domain in the *N-terminal* of the NS3 protein (the catalytic triad residues being His^51^—Asp^75^—Ser^135^) was discovered by sequence comparison (Fig. 5C) [62, 66–68]. A heterodimeric complex of NS2B-NS3 was found involved in the cleavage of protease-sensitive sites—NS2A-NS2B, NS2B-NS3, NS3-NS4A, and NS4B-NS5. This proteolytic processing step is vital in the assembly of the viral replicase complex and is a promising therapeutic target [62, 67]. An in-vitro study of the NS3 protein showed that it is not enzymatically active as a protease without the NS2B protein, contributed by the essential folding of the protein by NS2B. There have been no preclinical antiviral drug studies on the NS2B-NS3 complex despite the intensive research unveiling the structural properties of the molecule [69].
- **NS3 helicase** The structures of the *C-terminal* of the NS3 protease domain and NS3 helicase domain contain seven conserved motifs of NTPase and RNA helicases [44, 67]. The NS3 helicase facilitates the initiation of RNA synthesis and the melting of secondary structures. Resolving DNA duplexes formed during viral replication and separation of proteins bound to the viral genome is also regulated by NS3 helicase. RNA helicases have ATPase activity to facilitate the energy-dependent reaction of strand separation. Therefore, all NS3 helicases non-specifically hydrolyze nucleoside triphosphate to meet the energy requirement (hence known as “NTPase”) [70].

The use of NS3 helicase as an antiviral drug target has been challenging compared to the other non-structural proteins because of the limited understanding of the mechanism of its action. The selectivity of the compounds that can inhibit the ATP binding site being cytotoxic is another problem in using NS3 helicase as a drug target. Recently newer tests in high-throughput screening platforms based on DNA substrates have been developed to replace time-consuming traditional assays for screening helicases [67].

- **NS5 methyltransferase (MTase)** The *N-terminal* of the NS5 protein has methyltransferase activity (residues 1–262) and adopts the S-adenosylmethionine (SAM)—dependent methyltransferase fold, composed of four α helices surrounding central seven stranded β-sheets. The active site, containing a catalytic K61-D146-C180-N216 (KDCN) motif, is positioned in the center of the β-sheet (Fig. 5B) [71].
  NS5 MTase is an attractive drug target, and the focus on this drug target has increased in recent studies. One of the most crucial functions of NS5 MTase is the 5′-capping of nascent RNA. The *N-terminal* region of the NS5 protein methylates the 5′ guanine cap and the ribose 2′-OH position of the first transcribed nucleotide. Mutation studies on the NS5 MTase showed impaired viral replication, indicating that the enzyme plays an essential role in viral replication [72].
- **NS5 RNA-dependent RNA polymerase (RdRp)** The *C-terminal* of NS5 has polymerase activity (residues 273–900) and adopts a right-hand polymerase fold, consisting of fingers, palm, and thumb subdomains (Fig. 5E). The palm subdomain contains catalytic G662-D663-D664 (GDD) metal-binding motifs. JEV polymerase initiates RNA synthesis without the need for a primer [73].

NS5 RdRp at the *C-terminal* of the NS5 protein is one of the most promising and explored targets. This is primarily because of the absence of RNA-dependent RNA polymerase in humans. Nucleoside and non-nucleoside analogs have been extensively studied for targeting viral polymerase activity. The non-nucleoside compounds target the allosteric sites in the protein. Nucleoside analogs in the prodrug form are activated by phosphorylation to triphosphate. These active drug molecules inhibit the enzyme at the active site, restricting the chances of developing drug resistance; thus, are advantageous over non-nucleoside analogs [62, 73].

### Anti-Japanese encephalitis virus drugs

#### Nucleic acid-based antivirals

The discovery of the microRNA application in inhibiting viral genome transcription and translation was revolutionary in developing novel antiviral drugs. The exceptionally high specificity of such nucleic acid-based drug molecules makes them compelling candidates for therapeutic applications (Table 2). MicroRNA-based drug candidates examined that in-vitro and in-vivo have shown promising results by providing partial or complete protection in mice against JEV. These drugs have been targeted against genes coding for the Membrane, Envelope, Capsid, NS1, NS3, and NS5 proteins. However, a drawback of these therapeutics is that they must be administered simultaneously during or before JEV infection. They also lacked specificity to multiple strains or different genotypes [11, 74, 75].

**Table 2:** Anti-Japanese encephalitis virus drugs

Antiviral drug	Target/mechanism
Non-specific broad spectrum	
Interferon [11, 47, 76]	Interferon-stimulating genes—create an antiviral state and trigger the adaptive immune response
Aloe-emodin [77]	Interferon and interferon inducers
Ribavirin [39, 78–80]	Inosine monophosphate dehydrogenase—inhibits the synthesis of guanine nucleotides
Minocycline [42, 43]	Inhibits free oxygen radical generation resulting in reduced oxidative stress
Arctigenin [50, 52]	Anti-oxidative activity, anti-inflammatory activity
Fenofibrate [11, 81]	Anti-oxidative activity, anti-inflammatory activity
Aspirin indomethacin Sodium salicylate [82]	Cyclooxygenase inhibitors; modulates intracellular MAP kinase pathway following JEV infection
Rosmarinic acid [40, 82]	Anti-inflammatory and/or anti-apoptotic activity
Curcumin [11, 41]	Anti-oxidative activity Dysregulation of Ubiquitin–Proteasome system thus reducing the formation of new viral particles
Pentoxifylline [11, 79, 83]	Interferes with the assembly and release of the virus
Nitazoxanide [59, 84, 85]	Targets early mid-stage of viral replication Activates eIF2α
Diethyldithiocarbamate (DDTC) [86, 87]	Anti-oxidative activity
BCX4430 (Galidesivir) [88, 89]	C-nucleoside analog of adenosine
Luteolin [90, 91]	Inhibits E protein synthesis
Eflornithine [88, 90]	Enzyme-activated; an irreversible inhibitor of ornithine decarboxylase
Tubacin [92]	Inhibits Histone deacetylases (HDACs)
Astragali radix [35]	Non-specific mechanisms like anti-inflammatory activity
Anisomycin [82, 93]	Restores the function of the extracellular signal-regulated kinase (ERK) and suppresses JEV-induced cytotoxicity
Apoptozole [90, 94]	Inhibits the functioning of HSP70 protein
Erythrosin B [95]	Inhibits flavivirus NS2B-NS3 protease
Tilapia hepcidin (TH) 1–5 [96]	Anti-inflammatory and immunomodulatory activities
Dehydroepiandrosterone (DHEA) [93]	ERK activation and upregulation of MAPK pathway
Nucleic acid-based	
siRNA (Gene silencing)	Domain II of E-protein [97]
	NS5—coding Region [98]
	Capsid ‘C,’ Membrane ‘M,’ NS3—coding sequence [99]
	Envelope ‘E,’ NS3, NS4b—coding sequence [100]
	PrM, NS1, NS2A, NS2B, NS3, NS4A, NS4B, NS5—coding sequence [100]
	C, E, NS1, NS3, NS4B, NS5—coding sequence [99]
Peptide nucleic acids [101]	5′ UTR, 3′ UT of JEV genome
Morpholino oligomers [102]	5′ UTR, 3′ UT of JEV genome
Rin-expanded (“Fat”) nucleoside and nucleotide analogs [62, 103]	Inactivation of NS3 NTPase/helicase
DNAzymes [45]	3′ non-coding region of the JEV genome
shRNA [100]	E gene, C and NS4b1 gene
Replication cycle-based	
Proteoglycans (Heparin sulfate, Chondroitin sulfate) [11, 104–106]	Interferes with the attachment and entry of JEV
E-Protein Domain III binding peptide [11, 107, 108]	Inhibits the interaction of E-protein with the cell receptor
Surfactant modified nanoscale silicate platelet [11, 109]	Blocks viral adsorption to the cell
Indirubin [11, 110]	Inhibits virus attachment
Bovine lactoferrin [11, 111]	Binds to Heparin sulfate and prevents attachment
Griffithsin [11, 112]	Binds to E-protein and prevents attachment
2-Deoxy-d-glucose and 3-deazauridine [35]	Interference with the synthesis of JEV glycoprotein, DNA, and RNA
2-Methylnaphtho[2,3-b] furan-4,9-dione 2-(1-hydroxyethyl)-analog of naphtho[2,3-b] furan-4,9-dione 2-methyl-5(or 8)-hydroxyanalog naphtho[2,3-b] furan-4,9-dione [35]	Inhibits replication through inhibition of viral RNA and protein synthesis
Suramin [35]	Inhibits replication by blocking the production of viral E and NS3 proteins
Lactoferrin [35]	Inhibits JEV entry into the host cell by binding directly to the virus particle or to membrane-bound heparan sulfate
PI 88 [35]	Causes steric hindrance to JEV attachment to host cells; may possess immunomodulatory activity
MCPIP1 ribonuclease [11, 113]	Targets JEV genome. It has RNase activity and thus inhibits viral replication
Kaempferol [11, 114]	Binds to JEV frameshift site RNA (fsRNA) and inactivates the virus
Methyl-β-cyclodextrin [35]	Disrupts lipid raft formation by depleting cholesterol; inhibits replication and viral entry into host
Filipin III [35]	Chelates cholesterol; inhibits replication and viral entry into host
Bafilomycin A1 [35]	Inhibits vacuolar-type proton pump; inhibits pH-triggered membrane fusion of endocytosed JEV, thereby preventing replication
Dehydroepiandrosterone [35]	Modulates signaling pathways of extracellular signal-regulated protein kinase
N-methylisatin-beta-thiosemicarbazone derivative (SCH 16) [11, 78, 115]	Inhibits early translation
SK-12 protein [69, 116]	Inhibits NS2B-NS3 serine protease
Recombinant NS3 protein motif-IV [117]	Inhibits NS3 NTPase/helicase
N-nonyl-deoxynojirimycin (*N*N-DNJ), (Imino sugar derivative) [11, 44]	Inhibition of cellular glycoprotein processing α-glucosidase enzymes which leads to misfolding of viral proteins
FGIN-1–27, Cilnidipine [118]	Inhibits viral replication
Manidipine [11, 118]	Inhibits NS3 Helicase
Carrageenan (sulfated polysaccharide) [119]	Inhibits entry into host cells
Temoporfin [120]	Inhibition of the interactions between viral NS2B and NS3 proteins
NSC 12155 [121]	Inhibits NS5 methyltransferase activity
2F2 and 2H4 (monoclonal antibodies) [122]	Blocks attachment of the virus to its receptor
Lonafarnib [123]	Inhibits virus replication (viral entry)
Nitroxoline [123]	Inhibits virus replication
Cetylpyridinium chloride [123]	Inhibits virus replication
Cetrimonium bromide [123]	Inhibits virus replication
Hexachlorophene [123]	Inhibits virus replication
Belladonna [124]	Reduces caspase 3 and 8 enzymatic Activity NS3Protein and reduce its expression
Pokeweed antiviral protein [125]	Depurination of viral RNAs
Furanonaphthoquinone [126]	Inhibits the expression of viral proteins and also genomic RNA
Quercetin [127]	Intracellular virucidal activity, and Inhibits adsorption
Baicalein [127]	Intracellular virucidal activity, Inhibits adsorption
Amphotericin B [128]	Inhibits viral replication and/or the synthesis of viral proteins
In-silico modeling-based	
Ivermectin [11, 129]	NS3 Helicase Inhibitor
4-Hydroxy panduratin A [130]	NS2B-NS3 protease inhibitor
Bortezomib [131]	Targets JEV genome
Mycophenolate [132]	E-protein inhibitor

*C* capsid, *M* membrane, *E* envelope, *NS* non-structural, *prM* premembrane, *MAPK* mitogen-activated protein kinases, *JEV* Japanese Encephalitis Virus, *eIF2a* eukaryotic Initiation Factor 2a, *HSP70* Heat Shock Proteins 70, *ERK* extracellular signal-regulated kinases, *UTR* untranslated region, *NTPase* nucleoside triphosphatase, *DNA* deoxyribonucleic acid, *RNA* ribonucleic acid, *siRNA* small interfering RNA, *shRNA* short hairpin RNA, *DNAzymes* deoxyribozymes, *fsRNA* frameshift site RNA

Nucleic acid derivatives with heterocyclic bases as side chains and noncyclic peptide-like backbones were found to bind irreversibly to their complementary sequences with high specificity. These derivatives, called peptide nucleic acids (PNAs), inhibited viral translation. Numerous PNA-based drug candidates with varying specificity have been tested since their discovery. PNAs conjugated with cell-penetrating peptides were used for the study to ensure efficient uptake in cells. These PNAs targeted the untranslated regions (UTRs) of the viral genome and studied their anti-JE properties. The study revealed inhibition of the viral replication, which was attributed to the interference in genome cyclization induced by PNAs, highlighting their potential anti-JE activity [133].

#### Viral replication cycle-based antivirals

Theoretically, each step in the JEV replication cycle is a potential target for antiviral drug development. A compound targeting various stages—from the binding of the virus to the cellular receptors to genome replication, protein translation, maturation, and release—can inhibit the development of the virus. Numerous approaches were aimed at preventing the initial attachment of the virus to the cellular receptors. Heparan sulfate was found to be an essential cellular receptor and was considered a potential drug target. Cell-free heparin sulfate derivatives were studied in in-vitro and in-vivo systems, where these molecules exhibited partial protection against JEV infection [104]. RNA replication has been a potential drug target primarily because of the several factors governing it. The nuclease domain of a protein called Monocyte chemoattractant protein 1-induced protein 1 (MCPIP1) expressed antiviral activity against JEV in-vitro. MCPIP1 may target multiple JEV RNA sites and inhibit replication by interacting with RNase, RNA binding, and oligomerization [113, 134]. A plant-derived protein called Pokeweed antiviral protein isolated from *Phytolacca Americana* hindered viral replication in-vitro. This protein resulted in the depurination of the viral RNA. An in-vivo study involving the intraperitoneal administration of this antiviral protein to mice revealed partial protection against JE [9].

#### In-silico modeling-based antivirals

Recent advances in structural virology have helped in obtaining high-resolution images of viral proteins. These include NS3 C-terminal (NTPase/helicase catalytic domain) [135], E protein [64], NS5 [136], capsid protein [61], and NS2B-NS3 (JEV protease) [137]. Among these proteins, most antiviral drug candidates have been targeted against NS3, NS5, and E proteins primarily because of the essential role they play in the course of the infection [11].

## Antiviral susceptibility screening

The recent advancement in drug discovery has allowed researchers to avoid time-consuming trial-and-error methods, which, in most cases, prove costly. NMR and crystallography, computational advances in virtual screening, and the development of high-throughput screening (HTS) platforms have built the foundation of structure-based drug screening techniques. Rational drug screening (RDS) requires detailed knowledge of drug targets and drugs. It essentially involves a detailed analysis of the three-dimensional interaction between the target and the drug. A compound is selected and tested for its specific activity against the virus under consideration. This selection is based on the well-characterized understanding of the particular viral target. RDS technique requires a sound knowledge of structural chemistry and biology as the drug's activity depends on the chemical interactions between the viral target and the drug [138, 139].

### Computational screening studies

The development of a virtual screening platform was one of the most remarkable advancements that enabled the in-silico screening of multiple drugs with several targets simultaneously. The advances in structural chemistry like NMR spectroscopy techniques and crystallography, along with high-throughput protein purification, paved the way for the development of computational analysis of drug–protein and protein–protein interaction. Virtual screening (VS) techniques are mainly employed for ‘hit’ identification and ‘lead’ optimization. Computational screening is a direct and cost-effective way for rational drug screening compared to conventional high-throughput screening methods. Virtual screening involves ligand-based and structure-based methods. When the structural information is unavailable for targets, but the active ligand molecules are known, the ligand-based techniques are employed. Molecular docking is an example of structure-based drug design, where the complete structural details regarding the target and the drug molecule are known [140].

Molecular docking is a virtual screening technique that calculates the interaction energy between any two molecules. Docking employs algorithms like distance geometry methods, molecular dynamics, Monte Carlo simulation, fragment-based search, etc., to help understand the molecular interactions better. The best orientation for ligand binding to form a complex with a minimum energy of a molecule is found using the molecular docking technique. The ligand binds to the protein’s active sites, predicted by the search algorithms used [141].

Docking plays a vital role in computational drug design. Considerable efforts have been made towards improving docking algorithms due to the spectrum of molecular docking applications. The docking results are formalized by a statistical scoring function based on the interacting energy of the molecules and are called the docking score. Visualization of the bound ligand is done using visualizing tools like Pymol [141–143], Chimera, etc. [144, 145]. These 3D visualizations can help draw a better inference of the best fit of the ligand [138]. Several drugs have been screened for the Japanese encephalitis virus targets using various molecular docking programs like SYBYL, modeler, AutoDock, GOLD Suite (Genetic Optimization for Ligand Docking), etc. (Table 3).

**Table 3:** Docking programs used in virtual screening of compounds

Docking program	Antiviral drug	Target
SYBYL8.0 [132]	Compounds screened from Specs compound library	NS3 helicase/nucleoside
AutoDock 4.2 [11, 126]	4-hydroxy panduratin A	NS2B-NS3 protease
AutoDock 4 [83, 127]	Ivermectin	NS3 helicase
	Antiviral molecules and their analogs from the NCBI Pub-Chem compound database were identified for their drug-like properties using the Lipinski filter	E protein
AutoDock Vina [138, 140, 146]	Novel ligands developed with v1.2 software	NS3 protein
	43 bioactive bioflavonoids reported in *Azadirachta indica*	RdRp protein
modeler 9.10 [132]	Mycophenolate	E-protein
GOLD Suite 5.1 [142]	Compounds screened from the ZINC database	NS3 helicase/nucleoside
GLIDE [147]	Phytoconstituents of the Arisaema	JEV NS3 helicase, NS2B-NS3 protease, and NS5
Molegro Virtual Docker (MVD) [121]	Atropine and scopolamine	NS3 protein
iGEMDOCKv2.1 [148]	Aminoglycoside and Tetracycline group of compounds	NS3 helicase / nucleoside

*NS* non-structural, *E* envelope, *RdRp* RNA-dependent RNA polymerase, *JEV* Japanese encephalitis virus

### High-throughput screening

High-throughput screening (HTS) enables quick analysis of a series of chemical compounds. This technique is one of the most modern technologies employed in antiviral drug screening. This method helps in the characterization of affinities of biological structures. HTS includes a series of screening and assaying of biological modulators and effectors against specific targets. HTS assays can be used to screen various kinds of libraries, including drugs, peptides, proteins, and genomics. The principal objective of the HTS technique is to make a fast-track screening process for analyzing multiple drugs at a time. This technique enables screening at a rate that may exceed a few thousand compounds per day. This property of HTS is of utmost importance, because many novel compounds are being synthesized daily by combinatorial chemical synthesis. HTS technology significantly reduces the cost of research. Target identification followed by the preparation of the reagent and compound, assay development, and screening drug libraries is essential in HTS [149].

The compounds first undergo a primary screening, after which those compounds that give a positive result are considered as ‘hits’ and undergo secondary testing. The primary screening is less quantitative compared to traditional assays and calculates IC_50_ values (50% inhibitory concentrations of the compound). Secondary tests are usually biochemical tests that are more sensitive and specific to antiviral activity. HTS platform employs miniaturized cell-based assays or biochemical assays [149]. Drug libraries can be screened at a fast pace using assays that have been performed in an HTS platform to screen inhibitors of JEV infection [46]. Cell viability assays, such as Cell titer-Glo Luminescent Cell Viability Assay [117], MTT assay [150], LDH assay [102, 111], XTT assay [127, 150], FRET assay [118, 142], etc., have been performed in an HTS platform to analyze parameters such as cytotoxicity, antiviral activity, radical, and oxygen production.

### Plaque reduction neutralization assay

The plaque reduction assay (PRA), first described in the 1950s, is currently a standard method for in-vitro determination of antiviral drug susceptibility. PRA is the most commonly reported technique that sets a benchmark of comparison for many novel methods [151]. The design of the PRA measures the drug’s effects on the infectivity of the virus by plating the virus–drug mixture on a virus-susceptible cell line. Overlaying the cells with semi-solid media restricts the spread of the progeny virus [152]. Each virus particle multiplies under conditions that result in a localized area of infected cells or ‘plaque.’ The plaques are revealed either as areas of dead/destroyed cells detected by general cellular stains or as areas of infected cells detected by immunostaining [153]. The initial concentration of the virus in the stock is calculated from the number of plaques, and the total virus infectivity is calculated. The virus to be used for PRA is quantified initially by plaque assay. The antiviral drug is serially diluted to estimate the endpoint titers for each drug concentration [152]. However, PRA requires viral titration and prolonged incubation until the viral cytopathogenic effect is visible. This method is laborious, subjective, and time-consuming [151]. Plaque reduction assay being a standard phenotypic susceptibility test has been used for screening antiviral activity of molecules like curcumin [41], tripeptide NSK [154], griffithsin [112], and other drug candidates [105, 107].

### Other antiviral drug screening techniques

#### Quantitative real-time PCR (qPCR)

The qPCR is a PCR-based technique that integrates amplification of a target DNA sequence with quantification of its concentration. Quantification of nucleic acids by real-time PCR is done by two standard methods: relative quantification and absolute quantification. Absolute quantification employs a calibration curve plotted with the help of DNA standards to give the exact number of target DNA molecules. This type of quantification dictates that the amplification efficiency of the sample and the standard is the same. When internal reference genes are used to determine fold differences in the target gene expression, it is referred to as relative quantification [155–158]. The assay measures inhibition of viral DNA production by quantification of viral DNA using the TaqMan technology and assesses the effect of the drug on the virus [112, 155, 159].

#### Focus-reduction assay

The foci assay is a variation of the plaque assay, where the addition of a virus-specific primary and fluorescent-labeled secondary antibody is used to quantify the virus. After adsorption and gene expression, primary and secondary antibodies are added and incubated. The immobilized virus particles bound to the antibodies will form fluorescent foci, which can be observed under a microscope at an appropriate wavelength. A standardized amount of virus determined by the focus-forming assay is used for the focus reduction assay. The virus stock is titrated and is expressed in focus-forming units per milliliter [160, 161]. A variation of this technique uses insoluble dye bound to a secondary antibody instead of the fluorescent molecule. It is a practical cell-based antiviral drug susceptibility test [118]. A focus reduction assay demonstrated the application of chondroitin sulfate as an antiviral against JEV [105].

#### High content imaging

High content imaging is a type of HTS that employs virus-specific fluorescent labeling and a competent imaging platform to screen potential molecules for their antiviral activity. It is a relatively recent advancement in screening drug libraries and is highly efficient due to its accuracy in labeling the viral component. Immunofluorescent staining or construction of JEV virus expressing GFP reporter gene which generates fluorescent signal can be utilized for this assay [123, 162, 163]. Immunofluorescent staining involves the use of a JEV specific primary antibody (anti-JEV prM) and fluorescent-labeled secondary antibody (DyLight 488-labeled antibody). Cell monolayers infected with JEV (strain—AT31) are treated with the drug molecules and incubated. After 23 h of incubation, the cells are fixed and stained with the primary and fluorescent-labeled secondary antibody [162]. The reporter JEV genome is constructed using a wild-type JEV (pACYC-JEV-SA14) as the foundation. The eGFP (enhanced green fluorescent protein) gene is incorporated in the capsid region of the JEV genome, and the capsid protein of the resultant reporter virus emits fluorescent signals. The reporter virus can infect the cell monolayers and treat the drug molecules [123, 163].

The number of cells emitting fluorescence signals are then read by high-content imaging platforms such as Cell Voyager 7000S, PerkinElmer high-content screening system, and Operetta high-content imaging system. The recorded readings are then examined using analytical software (Harmony 3.5, GraphPad Prism 5.0) to quantify each drug molecule's infectivity and effectiveness [123, 162, 163].

#### Co-immunoprecipitation

Co-immunoprecipitation (Co-IP) is an efficient method used to study protein–protein interactions selectively. Proteins or ligands bound to a specific target protein are indirectly captured with the help of viral target protein-specific antibodies. Co-IP is an extension of immunoprecipitation. Other molecules such as the antiviral drug compound bound to the target protein by native interactions are also precipitated [164]. This modification of the co-immunoprecipitation technique that focuses on binding the drug molecule to the target viral protein enables it to be used as an efficient drug susceptibility screening tool [100, 159]. N-nonyl-deoxynojirimycin (NN-DNJ) interaction studies with cellular targets for studying anti-JEV properties were done using co-immunoprecipitation [44].

#### Virus yield assay

The virus yield reduction assay is a powerful technique for evaluating the efficacy of antiviral compounds; it is not routinely utilized as the process is quite laborious [165]. In this assay, different susceptible cells of the given virus are grown in 24-well plates and are infected with the virus in the presence of different concentrations of the compounds, at least two wells per concentration. After incubation in the cell, supernatants are collected, and the virus yields are determined by plaque formation in a susceptible cell line [148]. The antiviral activities of chemical compounds such as mycophenolic acid and CW-33 analogs were assessed using Virus Yield Assay [166, 167].

#### In-vivo antiviral studies using animal models

Animal models are frequently used to study antiviral drug candidates and can help better understand the mechanism of the drug intervention. In-vivo helps to understand the pathogenesis of an ongoing infection. They are also employed to get a much clearer picture of the cytotoxicity profile of the drug candidate in a clinical scenario. The mouse model has been the most preferred and widely used for studying viral encephalitis, employed for testing the effectiveness and safety of viral vaccines and therapeutics. A high degree of susceptibility to laboratory strains of JEV, similarity in disease presentation and virus tropism with humans, and availability of large numbers of animals for experimental purposes, etc., make the mouse model suitable for in-vivo studies [168]. Commonly observed neurological symptoms like poor pain response, piloerection, restriction of movements, body stiffening, limb paralysis, and whole-body tremor greatly resemble the symptoms in humans and are predictive of the disease. Thus, these signs often trigger intervention and other triggers such as the virus titer, virus antigen, and nucleic acid in brain samples. Molecular events caused by JEV infection in mouse brains have also been identified as triggers for intervention and are characterized by quantitative mass spectrometry studies. In infected mouse brains, several upregulated interferon-stimulated genes and induced inflammatory cytokines, such as IFN-γ, IL-6, TNF-α, and TGF-β in infected mouse brains, have served as biomarkers in studying JEV pathogenesis and the efficacy of treatment strategies [169–171].

Most of the studies on antivirals against JEV utilized neonates or adolescent (4- to 5-week-old) BALB/c mice, which, when infected with a lethal dose of JEV, exhibit a distinct pattern of infection. Consistent propagation of JEV in the brain to the levels necessary for antiviral drug screening was only achieved in neonate mice (1-week-old) inoculated intracerebrally or intravenously [172, 173]. An alternative was a mouse-adapted isolate of JEV (JEV-S3), which was used to develop a robust mouse model of JEV infection in adolescents (3–4 weeks old) C57BL/6 mice intraperitoneal route. The model developed clinical symptoms, with the virus entering and replicating vigorously in the brain. Triggers such as proinflammatory proteins were upregulated, eventually leading to death. Interestingly, BALB/c mice (3–4 weeks old) also exhibited identical susceptibility to JEV-S3 [169]. Therefore, the JEV-S3-infected animal model would help understand details of the JEV pathogenesis and screening antiviral molecules.

Drug candidates like griffithsin [112], tripeptide NSK [154], and imino sugars [44] were tested for their antiviral activity in mouse models. These procedures involved selecting 3–7-month-old mice (BALB/c, ICR), segregated into control and test groups. A non-cytotoxic concentration of the drug and a lethal dose of the virus was administered intraperitoneally in a peripheral challenge model. The drug was administered orally [44] as well as via injection intraperitoneally [112, 154]. After a period of observation for the various symptomatic triggers to manifest in controls, the mice were sacrificed randomly from each group, and brain samples were collected. These samples were further analyzed using plaque assay and western blot assay for quantification. The virus titer, which indicates the extent of virus multiplication in the drug's presence and absence, is a standard trigger utilized for in-vivo antiviral studies for JEV.

## Discussion

Japanese encephalitis (JE) caused by the JE virus (JEV) is one of the leading causes of viral encephalitis localized predominantly in the Asian region. Though JE is endemic to many parts of Asia, recently, several cases have been reported from areas such as northern Australia and the western Pacific region, where the threat was previously unknown [1]. Around 2 billion people inhabit areas experiencing significant risk of JEV [5]. Factors like population explosion, rapid globalization, migration, climatic shift, and large-scale escalation in rice cultivation have resulted in the spread and rise of JEV in the recent decade [5, 23]. Speculations that JEV can become a global pathogen in the future, causing worldwide pandemics, cannot be overlooked.

Currently, the efforts to control JE in India and other JEV endemic Asian countries are focused on widespread vaccination programs. The disease is preventable by vaccination and vaccination drives which have shown significant results [174]. However, the prevalence of neurological complications related to JEV remains significantly high, with death rate risks as substantial as 60% [140]. This points towards the urgent requirement to identify and develop specific therapeutic interventions to treat the infection. Although there have been many in-silico, in-vitro, and in-vivo studies dedicated to discovering anti-JEV compounds, the promise of a safe, effective, and affordable drug is afar. This challenge is particularly significant for JEV endemic countries, explicitly developing countries that suffer from low vaccine coverage.

Antiviral therapeutics indicate drugs or various therapies effective in treating virus infections in patients. Antiviral treatments must either inhibit the multiplication of the virus or be active within the infected cell [175]. Usually, antiviral drug therapies have been found to impede virus replication through different potential mechanisms. Therefore, therapeutics include drugs, which would work to clear the infection when administered earlier in infection, i.e., immediately post-exposure or sometimes even a few days later post-infection.

Many treatment options have been explored, ranging from interferon therapy to clinical trials with broad-spectrum antiviral drugs such as ribavirin. However, none of these have successfully treated JE [39, 47]. The shift in screening approaches from available antiviral drugs towards naturally occurring molecules was remarkable. Natural molecules such as rosmarinic acid (phenolic compound) and arctigenin showed protection in mice by markedly increasing proinflammatory mediators in the brain and decreasing JEV (GP78 strain)-induced neuronal apoptosis, caspase activity, and microglial activation. A broad-spectrum tetracycline antibiotic called minocycline also exhibited antiviral activity against JEV by decreasing JEV-induced neuronal apoptosis and proinflammatory activity in the brain in an in-vivo study [40, 50, 176].

*N*,*N*-methylisatin-β-thiosemicarbazone, a chemical derivative, and N-nonyl-deoxynojirimycin, a glucosidase inhibitor of the endoplasmic reticulum, completely inhibited JEV replication in-vitro [11, 78, 115]. Innovative ideas employing RNA interference techniques have also been tried. Before or after the viral challenge, a single intracranial administration of lentivirus-delivered short hairpin RNA or lipid-complexed small interfering RNA (siRNA) protected lethal encephalitis [97]**.**

A major challenge faced while studying many of these drugs was that these drugs were effective when administered before or immediately after the infection. Generally, it takes a considerable amount of time to onset symptoms in a clinical scenario, depending on incubation time, immune response, etc. Treatment is usually started after the disease has set in. Hence, there is a need for antiviral drugs effective against JEV prophylactically and therapeutically. Although in-vitro and in-silico studies can give information about a drug’s potential antiviral nature along with evidence of its cytotoxicity profile, a significant limitation associated with these study results is the varied translation of the effects in an in-vivo study. On the other hand, in-vivo studies using laboratory animals such as mice, and rabbits, mimicking a natural infection, may fail to predict the desired efficacy in humans.

JEV can affect the CNS in about 4–6 days after infection. Therefore, even with early diagnosis, there is a sufficient risk of CNS invasion by the virus before antiviral treatment is initiated [14]. Since the drugs cannot cross the blood–brain barrier, treatment in such cases will be ineffective. Therefore, an efficient drug delivery system is a requisite to ensure the activity of anti-JE drugs [177]. Lipid solubility of the BBB is an important feature that allows passive diffusion into the BBB. Specific chemical modification of the drug molecule may impart more permeability to the drug [178]. This modification limits the drug selectivity and distribution across the tissue. A prodrug and nanoparticles are alternate methods for better drug delivery and tissue specificity [177, 178].

Several drug molecules have been demonstrated to have significant anti-JEV activity in-vitro and in animal models; some of them widely used for treating other conditions [13]. The majority of these studies suggested a timeline of 5–6 days post-infection for initiating the treatment based on the prognosis of the disease in animal models. On average, 70–100% survival was observed with most of these therapeutics administered at the time of or shortly after JEV infection. However, molecules like minocycline, etanercept, and pentoxifylline proved effective when administered 5–6 days post-infection. [79, 179, 180]. Another example is the conferred protection provided by the anti-JEV monoclonal antibody on day five after infection [181]. None of the trials with therapeutics on Japanese encephalitis have successfully demonstrated a beneficial outcome to date. Besides, many approaches remain untested, which can be attributed to the delayed and incomplete understanding of the disease pathogenesis and treatment options in humans, despite advances in our comprehension of the disease mechanistic gathered from preclinical studies.

In the face of no effective drugs available for the treatment of JEV infection, it is essential to establish an efficient antiviral screening system to develop antiviral drugs. High-throughput screening (HTS) of antiviral drugs is a highly promising screening strategy, which usually employs two different approaches. One approach focuses on specific viral proteins targeted through in-vitro functional assays in a high-throughput platform. The latter uses in-vivo cellular antiviral assays in a high-throughput platform. Although the former gives a more simplistic setup, the second approach is broader and has lesser limitations. One efficient antiviral screening system was developed based on in-vivo cellular antiviral assays. It is involved the construction of reporter viruses from wild-type (WT) viruses, which can be used to isolate and understand antiviral drugs targeting the complete infection cycle. Rluc-JEV [123], Rluc-DENV [182], and eGFP-DENV [183] are examples of reporter flaviviruses developed for different research purposes.

Another example is an eGFP-JEV-based assay in a high-throughput platform employing an eGFP gene (eGFP-JEV) in a JEV reporter virus to screen 1443 compounds from an FDA-approved drug library. Sixteen drugs inhibiting JEV infection were identified, of which five drugs offered the potential for development as new therapies for the treatment of JEV infection [123]**.** The use of such novel systems in identifying JEV inhibitors may expedite the process of antiviral drug development considerably, thereby indicative of future advancements.

## Conclusion

Several new anti-flaviviral molecular targets and strategies have been defined with the advent of scientific research. Targeting viral proteins is one of the most attractive antiviral strategies and is vastly studied. These proteins exclusive to the virus allow the high specificity of the drug without affecting the host organism. The E and NS5 proteins of flaviviruses have proven promising targets for future drug design. Crystal structure determination of these proteins, especially the enzymatically active domains, provides information on their biochemical properties. In addition, the three-dimensional crystal structures of these proteins will facilitate the design of specific inhibitors with properties to alter the kinetics, subcellular localization, and regulation of the viral proteins. Focusing on the experimental studies to understand the physicochemical and biochemical properties of non-structural proteins of flaviviruses such as NS2B and NS3 also be highly useful. Crystallography-based models can also be used to study the protein substrate interaction. Advanced molecular biology knowledge and improved technological resources have rendered newer high-throughput anti-flaviviral drug screening pathways. Projects using the mega-computing power of the Autodock virtual docking program and several molecular dynamic programs have shown significant promise in anti-flaviviral drug research by helping identify drug-like molecules based on binding calculations mean-field molecular dynamics algorithms [165].

Different compounds isolated naturally or derived synthetically have exhibited targeted viral inhibition and significant antiviral properties. However, further research is quintessential to exploit the potential of these compounds to be developed into anti-JEV drugs. Despite extensive testing for anti-JEV activity through both in-vitro and in-vivo studies, none of these compounds have successfully exhibited a significant outcome in advanced studies. Accounting for the considerable percentage of JEV outbreaks occurring in developing countries, the need for effective, cheap, and readily available drugs is inevitable, besides increasing the vaccine coverage to overcome the challenge posed by JEV completely. Over the years, a better understanding of immunology, JEV pathogenesis, and replication mechanisms has aided in searching for novel anti-JE candidate drugs. However, awareness of the need for extensive research to deal with JE is still lacking worldwide. As the requirement for an anti-JEV drug is on the rise and as the search for antivirals for other flaviviruses accumulates research strategies for this work, several drug candidates can be expected to be soon evaluated in human clinical trials in the near future.

## Abbreviations

Asn: Asparagine
Asp: Aspartic acid
C: Capsid
Cys: Cysteine
DNA: Deoxyribonucleic acid
DNAzymes: Deoxyribozymes
E: Envelope
eIF2a: Eukaryotic initiation factor 2a
ERK: Extracellular-signal-regulated kinases
fsRNA: Frameshift site RNA
Gly: Glycine
Glu: Glutamic acid
HSP70: Heat Shock Proteins 70
His: Histidine
IFN-γ: Interferon Gamma
JEV: Japanese encephalitis virus
Lys: Lysine
M: Membrane
MAPK: Mitogen-activated protein kinases
NS: Non-structural
NTPase: Nucleoside triphosphatase
prM: Premembrane
RNA: Ribonucleic acid
RdRp: RNA-dependent RNA polymerase
Ser: Serine
siRNA: Small interfering RNA
shRNA: Short hairpin RNA
UTR: Untranslated region
WHO: World Health Organization
